# The ALT/AST Ratio as a Predictor of MASLD in Obese Children: A Comparative Analysis with Metabolic Indices Including HOMA-IR and TyG

**DOI:** 10.3390/jcm15114160

**Published:** 2026-05-28

**Authors:** Hilmi Onur Kabukçu, İlhan Hazer

**Affiliations:** 1 Department of Pediatrics, Private Kastamonu Anatolia Hospital, 37200 Kastamonu, Turkey; 2 Department of Pediatric Endocrinology, Kütahya Health Sciences University, 43100 Kutahya, Turkey; ilhan.hazer@ksbu.edu.tr

**Keywords:** MASLD, AT index, non-invasive biomarker

## Abstract

**Background/Objectives**: Metabolic dysfunction-associated steatotic liver disease (MASLD) is getting more prevalent in children with obesity, and simple and non-invasive biomarkers are needed for early detection. In this study, the discriminative performance of the ALT/AST ratio (AT index) for MASLD was evaluated in comparison with metabolic indices such as HOMA-IR and TyG derivatives. **Methods**: A total of 410 children with obesity aged 5–18 years were included in this prospective, multicenter cross-sectional study. Anthropometric measurements, laboratory data, and abdominal ultrasonography findings of the participants were evaluated. MASLD was diagnosed based on ultrasonographic findings. The discriminative performance of the indices was evaluated using ROC analysis, and factors independently associated with MASLD were assessed using multivariate logistic regression analysis. **Results**: BMI SDS, ALT, AT index, insulin, HOMA-IR, and TyG/ALT values were found to be significantly higher in the MASLD (+) group (*p* < 0.05). The AT index demonstrated statistically significant but modest discriminative ability for MASLD (AUC = 0.621; *p* < 0.001) and showed 70.5% sensitivity and 50.7% specificity at a cutoff value of >0.88. In the multivariate analysis, the AT index (OR: 3.401; 95% CI: 1.717–6.735; *p* < 0.001) and BMI SDS were identified as factors independently associated with MASLD.HOMA-IR and prolactin-related indices were not found to be significant as independent predictors. **Conclusions**: The ALT/AST ratio is a simple and accessible biomarker associated with MASLD in children with obesity. Although its discriminative performance is modest, its association with hepatic involvement and metabolic dysfunction suggests that it may provide supportive exploratory information in the evaluation of MASLD. However, further validation studies are required before broader clinical application.

## 1. Introduction

Childhood obesity has become a major public health problem worldwide in recent years, and a marked increase in associated metabolic complications has been observed. Among these complications, steatotic liver disease is the most common chronic liver disease in children and adolescents [1]. Traditionally referred to as “non-alcoholic fatty liver disease (NAFLD),” this condition has been redefined in the current literature under the umbrella of “steatotic liver disease (SLD),” more specifically as “metabolic dysfunction-associated steatotic liver disease (MASLD)” [2]. Recent meta-analyses show that the prevalence of MASLD in the pediatric population is about 13% in the general population and increases to up to 38% in the population of children with obesity. This shows the strong association of the disease with obesity and metabolic disorders [3].

The pathophysiology of pediatric MASLD is largely associated with insulin resistance, dyslipidemia, and systemic metabolic imbalance [4]. While liver biopsy is accepted as the gold standard for diagnosis, its routine use in pediatric patients is limited due to its invasive nature. Ultrasonography is the most commonly used imaging method for the evaluation of pediatric MASLD in the diagnostic approach. It is widely preferred in clinical practice since it is non-invasive, easily accessible, and cost-effective. However, the limited sensitivity of ultrasonography in detecting early-stage hepatic steatosis and its inability to provide a quantitative assessment are important disadvantages [5]. Therefore, investigating non-invasive biochemical markers that can be used together with ultrasonography has great clinical importance.

In recent years, metabolic indices derived from routine laboratory parameters have gained attention in this field. In particular, the triglyceride-glucose (TyG) index and its derivatives, especially the TyG/ALT index, have been suggested as practical markers reflecting insulin resistance and hepatic steatosis. Recent studies show that these indices may be associated with both the presence and severity of hepatic steatosis in the pediatric population [6,7]. In addition to metabolic markers, the relationship between hormonal factors such as prolactin and metabolic diseases is also being investigated. Prolactin is known to play a role in glucose metabolism, lipid regulation, and energy balance. However, the literature shows conflicting findings regarding the relationship between prolactin levels and pediatric MASLD [8,9]. In addition, simple liver enzyme-based ratios such as the ALT/AST ratio have been evaluated as potential indicators of hepatic involvement in metabolic disorders. These markers are particularly useful in clinical practice because they are easy to obtain and inexpensive. However, their exact relationship with MASLD in children with obesity is still not fully understood [10,11].

Studies evaluating metabolic indices together with hormonal markers in pediatric obese patients remain limited despite the existing literature. In particular, the combined assessment of ALT/AST ratios, prolactin levels, and TyG-based indices represents an area that has not been sufficiently studied in predicting MASLD. Therefore, this study aimed to investigate the relationship between MASLD and ALT/AST ratios, prolactin levels, and triglyceride-glucose-based indices in children with obesity. By evaluating these parameters together, it was aimed to identify non-invasive markers that may contribute to the exploratory evaluation of hepatic involvement and metabolic alterations associated with pediatric MASLD.

## 2. Materials and Methods

### 2.1. Study Design and Participants

This study is a prospective, multicenter cross-sectional study conducted at two centers between November 2024 and May 2025. The study was carried out at the Pediatric Endocrinology Outpatient Clinic of Kutahya Health Sciences University Kutahya City Hospital and the Pediatric Outpatient Clinic of Kastamonu Training and Research Hospital. Children aged 5–18 years who were diagnosed with obesity at admission (body mass index Z score >2 according to age and sex) were included in the study [12]. A total of 410 children with obesity were included in the study.

### 2.2. Exclusion Criteria

Obesity due to endocrine or genetic diseases (e.g., Cushing syndrome, syndromic obesity), thyroid diseases, use of hepatotoxic drugs, presence of chronic liver disease other than MASLD, viral hepatitis, autoimmune liver diseases, metabolic/genetic liver diseases (e.g., Wilson disease, alpha-1 antitrypsin deficiency), malnutrition, alcohol use (in adolescents), secondary hepatosteatosis due to drugs or toxins, uncontrolled metabolic diseases, and individuals with severe systemic disease were excluded from the study.

### 2.3. Data Collection and Measurements

Demographic data (age, sex) and anthropometric measurements of the patients were recorded. Blood samples were taken from all participants after at least 8 h of fasting, and plasma glucose, insulin, total cholesterol, triglycerides, HDL cholesterol, LDL cholesterol, aspartate aminotransferase (AST), alanine aminotransferase (ALT), HbA1c, thyroid-stimulating hormone (TSH), free thyroxine (fT4), and prolactin levels were measured. All laboratory analyses were performed in both centers using calibrated standard automated analyzers and following the same laboratory procedures. Plasma glucose concentrations were analyzed using fasting blood samples collected in fluoride-containing tubes and processed according to standard laboratory procedures [13]. To assess insulin resistance, the HOMA-IR index was calculated by dividing the product of fasting insulin (µIU/mL) and fasting glucose (mg/dL) by 405 [14]. The triglyceride-glucose (TyG) index was calculated using the formula ln [triglyceride (mg/dL) × glucose (mg/dL)/2] and was used as a practical indicator of insulin resistance [15]. In addition, the TyG/ALT index, triglyceride/HDL ratio, LDL/HDL ratio, and ALT/AST ratio (AT index) were calculated and analyzed as markers of metabolic and hepatic risk [16,17]. Upper abdominal ultrasonography was performed in all participants to evaluate hepatic steatosis. Ultrasonographic examinations were performed at two tertiary centers using the same ultrasound system (Siemens Acuson S3000 (Siemens Healthcare GmbH, Erlangen, Germany)) with a 6C1 convex transducer by experienced radiologists with at least 15 years of experience in abdominal ultrasonography. The radiologists were blinded to the laboratory findings of the participants. Hepatic steatosis was graded according to standard ultrasonographic criteria based on hepatic echogenicity, visualization of intrahepatic vascular structures, and posterior beam attenuation. Steatosis severity was classified as grade 0 (absent), grade 1 (mild), grade 2 (moderate), or grade 3 (severe). The diagnosis of MASLD was established by demonstrating hepatic steatosis on ultrasonography and excluding secondary causes [18]. However, because subgroup analyses according to steatosis grades did not yield statistically meaningful results and some subgroups had limited sample sizes, statistical analyses were performed using a binary classification (MASLD present vs. absent). Final statistical analyses were performed using a complete dataset including only participants with fully available clinical, laboratory, anthropometric, and ultrasonographic data. Therefore, no missing data imputation method was required.

### 2.4. Statistical Analysis

Statistical analyses were performed using the Statistical Package for Social Sciences (SPSS Inc., Chicago, IL, USA) version 20.0. The distributional characteristics of continuous variables were assessed not solely through formal normality tests, but also through evaluation of histograms, Q–Q plots, boxplots, skewness-kurtosis measures, the presence of outliers, and sample size considerations. Homogeneity of variances was assessed using Levene’s test. Continuous variables with normal distribution were presented as mean ± standard deviation (minimum–maximum), whereas non-normally distributed variables were presented as median (IQR; 25th and 75th percentiles) [19]. For comparison of measurement values between two groups, the Student’s *t*-test was used when normal distribution was met, and the Mann–Whitney U test was used when the assumption of normal distribution was not met. Relationships between variables were analyzed using the Spearman rho correlation coefficient (ρ). For correlation analysis, a coefficient <0.390 was considered a weak relationship, 0.400–0.590 a moderate relationship, and >0.600 a strong relationship. In addition, receiver operating characteristic (ROC) analysis was performed to assess and compare the discriminatory performance of selected biomarkers for distinguishing MASLD status and to determine the optimal cutoff value for each parameter. Optimal cutoff values were determined using the maximum Youden index (sensitivity + specificity − 1). The area under the curve (AUC) was interpreted as follows: <0.5, not useful; 0.5–0.7, weak; 0.7–0.9, good; and 0.9–1.0, excellent. AUC values were reported together with 95% confidence intervals (95% CI). Binary logistic regression analysis was performed to identify factors independently associated with MASLD. Variables considered clinically relevant were first evaluated using univariable logistic regression analysis, and variables with *p* < 0.10 were considered candidates for the multivariable model. Multivariable logistic regression analysis was performed using the Enter method. During model construction, clinical relevance and potential multicollinearity among candidate independent variables were considered. Multicollinearity was formally assessed using variance inflation factor (VIF), tolerance statistics, and condition indices before multivariable model construction. Variables demonstrating substantial biological or mathematical overlap were carefully evaluated to avoid redundancy and unstable coefficient estimates. Results were presented as regression coefficients (B), standard error (SE), odds ratios (OR), and 95% confidence intervals (95% CI). Model fit was assessed using the Hosmer–Lemeshow goodness-of-fit test, and explanatory performance was evaluated using the Nagelkerke R^2^ coefficient. Results were presented as odds ratio (OR) and 95% confidence interval (CI) [20,21,22]. All statistical tests were two-tailed, and *p* < 0.05 was considered statistically significant.

### 2.5. Ethical Approval

This study was evaluated and approved by the Non-Interventional Clinical Research Ethics Committee of Kutahya Health Sciences University (decision no: 2024/13-24, date: 18 November 2024). The rationale, objectives, and methods of the study were reviewed by the ethics committee, and it was unanimously concluded that there was no ethical or scientific objection to conducting the study at the specified centers. The study was conducted in accordance with the principles of the Declaration of Helsinki, informed consent was obtained from the parents of all participants included in the study, and patient data were evaluated in accordance with confidentiality principles.

## 3. Results

A total of 410 children with obesity were included in the study. Of the participants, 57.1% were female (n = 234) and 42.9% were male (n = 176). The proportion of males in the MASLD (+) group (48.7%) was statistically significantly higher than in the MASLD (−) group (37.8%) (*p* = 0.026). The median age of the participants was 158.0 months (IQR: 122.0–190.0), and no significant difference was found between the groups in terms of age (*p* = 0.613).

Individuals in the MASLD (+) group had significantly higher BMI SDS, ALT, AT index (ALT/AST), insulin, HOMA-IR, and TyG/ALT index values (all *p* < 0.05). However, no statistically significant differences were observed between the groups regarding AST, triglyceride, HDL, LDL, TG/HDL ratio, LDL/HDL ratio, glucose, TyG index, prolactin, prolactin/ALT ratio, or HbA1c (all *p* > 0.05). Median prolactin levels were 11.00 (7.59–16.08) in the MASLD (+) group and 10.83 (7.24–15.30) in the MASLD (−) group (*p* = 0.531). Similarly, the prolactin/ALT ratio did not differ significantly between the groups (*p* = 0.150). HbA1c levels were also comparable between groups (5.50 [5.30–5.70] vs. 5.50 [5.20–5.70], *p* = 0.458) (Table 1).

The correlation analysis between the degree of hepatic steatosis defined by ultrasonography (USG) and biomarkers is presented in Table 2. A low-level, positive, and statistically significant correlation was found between USG findings and the AT index (ρ = 0.234, *p* < 0.001). Similarly, low-level but statistically significant positive correlations were observed between the degree of hepatic steatosis and the TyG/ALT index (ρ = 0.181, *p* < 0.001) and the HOMA-IR index (ρ = 0.217, *p* < 0.001). In contrast, no statistically significant relationship was found between the degree of hepatic steatosis defined by USG and TG/HDL, LDL/HDL, and TyG indices, as well as prolactin level and prolactin/ALT ratio.

When the relationships between indices were evaluated, a strong positive correlation was found between the AT index and the TyG/ALT index (ρ = 0.767, *p* < 0.001). The AT index also showed low-level positive correlations with TG/HDL (ρ = 0.143, *p* = 0.004), LDL/HDL (ρ = 0.175, *p* < 0.001), TyG index (ρ = 0.106, *p* = 0.032), and HOMA-IR (ρ = 0.282, *p* < 0.001). In contrast, a moderate negative and statistically significant correlation was found between the AT index and the prolactin/ALT index (ρ = −0.433, *p* < 0.001). The TyG/ALT index showed a low-level positive correlation with HOMA-IR (ρ = 0.134, *p* = 0.006) and a weak negative correlation with prolactin (ρ = −0.138, *p* = 0.005), while it showed a strong negative correlation with the prolactin/ALT ratio (ρ = −0.679, *p* < 0.001). In addition, a high-level positive correlation was found between prolactin level and the prolactin/ALT ratio (ρ = 0.779, *p* < 0.001) (Table 2).

**Figure 1:**
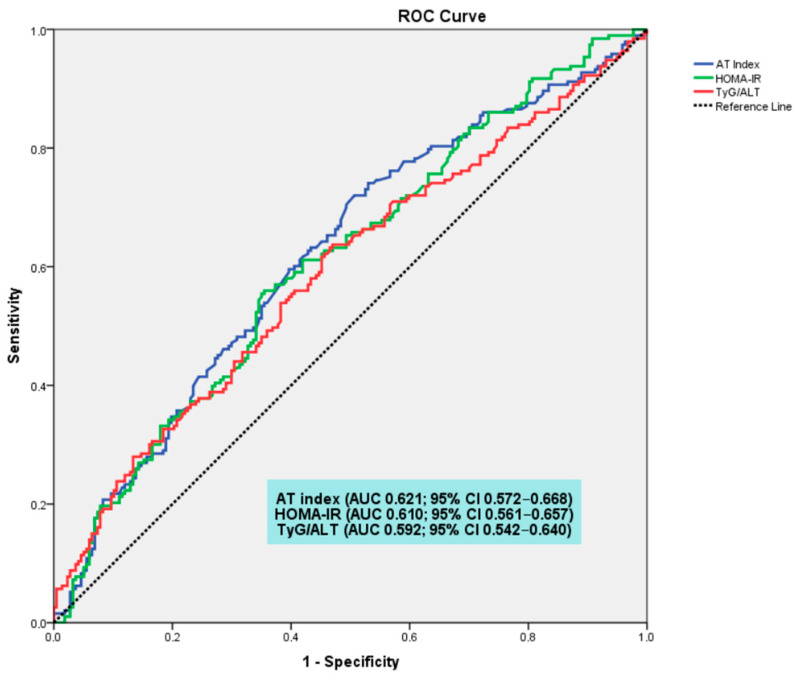
ROC Curves of Selected Biomarkers for Discriminating MASLD Status. Receiver operating characteristic (ROC) curves demonstrating the discriminative performance of the AT index, HOMA-IR, and TyG/ALT ratio for MASLD status. AUC: area under the curve; MASLD: metabolic dysfunction-associated steatotic liver disease; HOMA-IR: homeostasis model assessment of insulin resistance; TyG: triglyceride-glucose index; ALT: alanine aminotransferase.

The ROC analysis results of biomarkers evaluated for discriminating MASLD status are presented in Table 3, and the corresponding ROC curves of the principal biomarkers are shown in Figure 1. Sensitivity, specificity, AUC (area under the curve) values, 95% confidence intervals, and optimal cutoff values were assessed for indices used to distinguish MASLD (+) and MASLD (−) groups.

The AT index yielded an AUC of 0.621 (95% CI: 0.572–0.668; *p* < 0.001), indicating statistically significant but modest discriminatory performance for distinguishing MASLD status. At the optimal cutoff value of >0.88, the sensitivity was 70.4% (95% CI: 65.1–78.2) and specificity was 50.7% (95% CI: 42.0–55.7), with a positive likelihood ratio of 1.43 (95% CI: 1.20–1.65), a negative likelihood ratio of 0.58 (95% CI: 0.44–0.75), and a Youden index of 0.2116. The TyG/ALT index also demonstrated statistically significant but limited discriminatory ability, with an AUC of 0.592 (95% CI: 0.542–0.640; *p* = 0.001). At a cutoff value of >78.22, sensitivity was 63.8% (95% CI: 56.5–70.5) and specificity was 53.0% (95% CI: 45.7–59.3), with a positive likelihood ratio of 1.36 (95% CI: 1.13–1.60) and a negative likelihood ratio of 0.68 (95% CI: 0.55–0.87). Similarly, HOMA-IR showed modest discriminatory performance, with an AUC of 0.610 (95% CI: 0.561–0.657; *p* < 0.001). The optimal cutoff value was >4.37, corresponding to sensitivity of 55.5% (95% CI: 48.1–62.6) and specificity of 65.0% (95% CI: 57.8–70.9), with a positive likelihood ratio of 1.58 (95% CI: 1.25–1.95) and a negative likelihood ratio of 0.69 (95% CI: 0.57–0.83). TG/HDL, LDL/HDL, TyG, prolactin, and the prolactin/ALT ratio did not demonstrate statistically significant discriminatory performance for MASLD (all *p* > 0.05) (Table 3).

Multivariate logistic regression analysis to identify factors independently with the presence of MASLD is shown in Table 4. Multicollinearity diagnostics demonstrated no evidence of problematic collinearity among candidate variables included in the multivariable model (all VIF values <1.3, tolerance values >0.78, and condition indices <14). The AT index and BMI SDS were identified as factors independently associated with MASLD. Each unit increase in the AT was associated with 3.4-fold higher odds of MASLD (Odds Ratio (OR): 3.401; 95% CI: 1.717–6.735; *p* < 0.001), while an increase in BMI SDS increased the risk by approximately 1.7-fold (OR: 1.745; 95% CI: 1.301–2.340; *p* < 0.001). In contrast, HOMA-IR (*p* = 0.305) and the prolactin/ALT ratio (*p* = 0.085) were not found to be significant as independent predictors (Table 4).

## 4. Discussion

In this study, the ALT/AST ratio (AT index) emerged as a statistically significant biomarker associated with MASLD in children with obesity. The higher AT index in the MASLD (+) group suggests that this parameter may reflect early hepatic involvement. The AUC value of the AT index was 0.621 (95% CI: 0.572–0.668; *p* < 0.001) in the ROC analysis, showing statistically significant but clinically limited to moderate discriminative power. Although statistically significant, this AUC value indicates only modest discriminative ability and suggests that the AT index should be interpreted as a supportive rather than definitive diagnostic marker. The cutoff value of >0.88 provides a practical threshold, with 70.5% sensitivity and 50.7% specificity, especially for screening purposes. One of the key findings of our study is that the AT index was independently associated with MASLD in the multivariate analysis. The significant association between the AT index and MASLD in multivariate analysis suggests that this parameter may reflect hepatic involvement independently of some metabolic parameters; however, this association should not be interpreted as evidence of strong individual-level predictive performance. When the studies in the literature are reviewed, in a study including 4753 participants using NHANES 2017–2018 data, each unit increase in the AT index was reported to increase the likelihood of developing MASLD by 3.6-fold, and in that study, the cutoff value for the AT index was >1.3 in the general population and >0.86 in individuals under 20 years of age [11]. The findings of this study, which is considered one of the most comprehensive studies in the literature in this field, are consistent with our results and support their reliability. In another study conducted on 205 patients diagnosed with MASLD to evaluate liver fibrosis, an increased AST/ALT ratio was reported to be associated with advanced liver fibrosis, with a cutoff value of >0.94 [23]. Although this may seem contradictory to our findings at first glance, it is actually related to the stage-dependent progression of liver injury. While ALT predominance is typical in early hepatocellular injury, such as hepatosteatosis, advanced fibrosis is characterized by a relative increase in AST due to mitochondrial dysfunction, leading to an increased AST/ALT ratio [24]. In other words, the increase in the AT index observed in our study can be considered a marker of early hepatocellular injury. In light of all these findings, the AT index should be considered not as a standalone diagnostic tool, but as a complementary marker supporting clinical evaluation. In addition, the limited number of studies evaluating this relationship in the pediatric population increases the originality of this study.

In the ROC analysis results of the study, the performance of the AT index showed a notable difference compared to other metabolic indices. The lack of significance of TG/HDL, LDL/HDL, and TyG indices indicates that these parameters are limited in the differential diagnosis of MASLD. In contrast, although TyG/ALT (AUC = 0.592; *p* = 0.001) and HOMA-IR (AUC = 0.610; *p* < 0.001) were found to be significant, they yielded results close to but not exceeding the performance of the AT index. The absence of significant differences in lipid parameters between the groups suggests that classical lipid abnormalities may not always be evident in the early stages of MASLD. This finding is consistent with previous studies reporting that dyslipidemia may not be a dominant feature in the early stage of hepatic steatosis, especially in pediatric populations [8,24]. Current guidelines published by the American Association for the Study of Liver Diseases emphasize that serum transaminases, especially alanine aminotransferase, may be more sensitive indicators of early hepatocellular injury; in contrast, lipid changes usually become evident in the later stages of the disease [23]. Similarly, the North American Society for Pediatric Gastroenterology, Hepatology, and Nutrition states that lipid parameters alone have limited diagnostic value in the early diagnosis of pediatric MASLD [8]. Various methods are used to assess insulin resistance (IR) in childhood. TyG has been proposed as an alternative marker for assessing IR in children with MASLD. In two recently published studies investigating the relationship between TyG and MASLD in childhood and adolescence, TyG demonstrated superior performance compared with HOMA-IR [25,26]. Nevertheless, our findings partially support the existing literature regarding the relationship between TyG-related indices and MASLD. Although TyG was not identified as an independent predictor in our analysis, its association with MASLD-related metabolic alterations suggests that it may still have potential clinical relevance. The superiority of TyG over HOMA-IR in reflecting IR in patients with MASLD may be explained by several mechanisms. First, triglyceride accumulation in hepatocytes is considered one of the key mechanisms contributing to hepatocellular injury. Second, the liver plays a critical role in glucose metabolism through gluconeogenesis and glycogenolysis. As TyG reflects the interaction between these metabolic pathways, it may better reflect insulin resistance and metabolic dysfunction in patients with MASLD. Genetic and environmental factors play a role in the etiology of childhood MASLD. Recent studies have shown that the PNPLA3, TM6SF2, GPAM, and TRIB1 genes may be implicated in the pathogenesis of MASLD; however, studies on the genetic basis of MASLD remain insufficient. The fundamental pathogenic mechanism involves increased triglyceride accumulation in hepatocytes. In addition to the afore-mentioned genetic and environmental factors, increased IR, adipokines secreted from adipocytes, and alterations in the gut microbiota also contribute to the development of MASLD [27]. While insulin resistance represents a central component of MASLD pathophysiology, HOMA-IR did not retain independent predictive significance in the multivariate analysis. This suggests that although insulin resistance makes an important contribution, markers that directly reflect hepatic injury may be more decisive clinically. Similarly, some studies have reported that the independent predictive power of metabolic indices may be limited [3,8,24]. In light of these findings, our study suggests that the AT index may provide complementary information alongside metabolic parameters in the assessment of MASLD risk; however, its diagnostic performance remains limited. These results indicate that although metabolic dysfunction and hepatic injury are related, they may have different predictive strengths in multivariate models.

In our study, the lack of a significant association between prolactin levels and MASLD may be partly explained by physiological variability related to pubertal status. Prolactin secretion is regulated by the hypothalamic–pituitary axis and tends to increase, especially during puberty, due to rising estrogen levels. Therefore, in the pediatric population, hormonal fluctuations related to age and pubertal status may lead to inter-individual variability in prolactin levels [28]. Studies in the literature examining the relationship between prolactin and hepatic steatosis report conflicting results. While some studies have shown an association between low prolactin levels and increased MASLD risk, others have not confirmed this relationship [8,9]. These differences are thought to be mainly due to pubertal status and hormonal variability, especially in the pediatric population [8,20].

Although ultrasonography is considered an operator-dependent imaging modality, several methodological precautions were taken in the present study to minimize variability. Ultrasonographic examinations were performed using the same imaging system and transducer at both centers by experienced radiologists blinded to laboratory findings. In addition, standardized ultrasonographic grading criteria were applied for the evaluation of hepatic steatosis. These measures likely increased the consistency and reliability of the ultrasonographic assessments.

It is known that the prevalence of pediatric MASLD has increased markedly in recent years. Recent meta-analyses report that MASLD is seen in approximately 13% of the general population and 38% of children with obesity [3]. This situation increases the need for non-invasive and easily applicable biomarkers associated with hepatic involvement and metabolic dysfunction in pediatric MASLD. Although the diagnostic performance of the AT index is limited, its persistence as an independent predictor in multivariate analysis suggests that this parameter may be more valuable as a biomarker reflecting risk stratification and disease burden rather than as a diagnostic test. The fact that the AT index can be obtained from routine laboratory tests may increase its practicality as a supportive biomarker in clinical settings. Our study has some limitations. First, it was conducted in two centers, which may limit generalizability. The use of ultrasonography in the diagnosis of MASLD may lead to limited sensitivity, especially in detecting early-stage steatosis. Although hepatic steatosis was classified according to ultrasonographic severity grades, the relatively limited number of patients within some steatosis subgroups restricted the statistical power of severity-based analyses. In addition, although ultrasonographic evaluations were performed using the same imaging system by experienced radiologists blinded to laboratory findings, interobserver variability inherent to operator-dependent imaging methods cannot be completely excluded. In addition, although standardized protocols were used at both centers, potential inter-center differences in patient characteristics and referral patterns cannot be completely excluded. The inability to fully control pubertal status and lifestyle factors is among the other important limitations. This study did not include formal internal validation procedures, such as bootstrap resampling or cross-validation, nor external validation in an independent cohort. Therefore, the reported discriminative performance estimates and ROC-derived cut-off values should be interpreted as exploratory findings. Further validation in independent pediatric populations is required before broader clinical application.

In conclusion, this study demonstrates that the ALT/AST ratio is significantly associated with MASLD in children with obesity and may serve as a simple and accessible complementary biomarker associated with MASLD. However, given its modest diagnostic performance, the AT index should not be considered a standalone diagnostic tool. Furthermore, the absence of formal internal or external validation warrants cautious interpretation of the reported discriminative performance and ROC-derived cut-off values. Further prospective, longitudinal, and externally validated studies are needed to better clarify its clinical utility and relationship with disease progression.

## Figures and Tables

**Table 1:** Biomarkers That Can Be Used to Diagnose MASLD in Patients with Obesity.

Parameter	Total (n = 410)	MASLD (−) (n = 217)	MASLD (+) (n = 193)	*p*
**Gender**				
**Male,** *n (%)*	176 (42.9)	82 (37.8)	99 (48.7)	**0.026 ^K^**
**Female,** *n (%)*	234 (57.1)	135 (62.2)	94 (51.3)	
**Age (month),** *median (IQR)*	158.00 (122.00–190.00)	158.00 (120.00–191.00)	158.00 (132.00–188.00)	0.613 ^M^
**BMI SDS,** *median (IQR)*	2.70 (2.31–3.14)	2.62 (2.15–3.04)	2.81 (2.47–3.28)	**<0.001** ^M^
**ALT,** *median (IQR)*	18.00 (14.00–25.00)	17.00 (13.00–22.40)	19.30 (14.70–28.00)	**0.002** ^M^
**AST,** *median (IQR)*	19.60 (16.00–24.20)	19.60 (16.00–24.00)	19.60 (16.00–25.00)	0.500 ^M^
**AT index (ALT/AST),** *median (IQR)*	0.96 (0.77–1.22)	0.88 (0.73–1.13)	1.05 (0.83–1.30)	**<0.001**
**TG**	98.20 (73.00–139.40)	95.70 (68.80–131.80)	100.80 (76.50–145.10)	0.660 ^M^
**HDL,** *mean ± SD (min–max)*	46.08 ± 9.82 (20.0–78.0)	46.59 ± 9.62 (20.00–78.00)	45.51 ± 10.03 (24.0–77.0)	0.268 ^T^
**TG/HDL,** *median (IQR)*	2.21 (1.52–3.27)	2.21 (1.47–3.07)	2.20 (1.58–3.43)	0.329 ^M^
**LDL,** *mean ± SD (min–max)*	100.10 ± 27.74 (30.0–224.0)	100.25 ± 25.54 (36.0–169.0)	99.93 ± 30.09 (30.0–224.0)	0.905 ^T^
**LDL/HDL,** *median (IQR)*	2.19 (1.74–2.72)	2.16 (1.74–2.67)	2.24 (1.75–2.83)	0.403 ^M^
**Glucose,** *median (IQR)*	87.00 (82.00–93.00)	87.00 (82.00–93.00)	88.00 (83.00–93.00)	0.298 ^M^
**TyG index,** *mean* *± SD (min–max)*	4.55 ± 0.26 (3.72–5.86)	4.54 ± 0.24 (3.72–5.30)	4.56 ± 0.28 (3.99–5.86)	0.351 ^T^
**TyG/ALT index,** *median (IQR)*	81.45 (62.34–115.00)	76.84 (59.43–104.79)	87.79 (65.85–128.32)	**0.001** ^M^
**Insulin,** *median (IQR)*	18.95 (12.24–27.27)	16.90 (11.28–24.24)	21.23 (13.30–30.46)	**<0.001** ^M^
**HOMA-IR,** *median (IQR)*	4.06 (2.54–5.89)	3.62 (2.31–5.49)	4.64 (2.93–6.71)	**<0.001** ^M^
**Prolactin,** *median (IQR)*	10.90 (7.32–15.50)	10.83 (7.24–15.30)	11.00 (7.59–16.08)	0.531 ^M^
**Prolactin/ALT index,** *median (IQR)*	0.56 (0.35–0.97)	0.60 (0.37–1.01)	0.54 (0.32–0.95)	0.150 ^M^
**HbA1c,** *median (IQR)*	5.50 (5.20–5.70)	5.50 (5.20–5.70)	5.50 (5.30–5.70)	0.458 ^M^

ALT: Alanine aminotransferase, AST: Aspartate aminotransferase, AT index (ALT/AST): Alanine aminotransferase/aspartate aminotransferase ratio, Glucose: Fasting plasma glucose, HbA1c: Hemoglobin A1c, HDL: High-density lipoprotein, HOMA-IR: Homeostasis model assessment of insulin resistance, Insulin: Fasting insulin level, LDL: Low-density lipoprotein, LDL/HDL: Low-density lipoprotein/high-density lipoprotein ratio, MASLD: Metabolic dysfunction-associated steatotic liver disease, TyG index: Triglyceride-glucose index, BMI SDS: Body mass index standard deviation score. ^M^; Mann–Whitney U test, ^T^; Student’s *t*-test, ^K^; Chi-square test.

**Table 2:** Correlation Analysis Between MASLD Indices and USG Findings.

	USG Findings	AT Index (Rho, *p*)	TG/HDL (Rho, *p*)	LDL/HDL (Rho, *p*)	TyG Index (Rho, *p*)	TyG/ALT Index (Rho, *p*)	HOMA-IR Index (Rho, *p*)	Prolactin (Rho, *p*)	Prolactin/ALT Index (Rho, *p*)
**USG Findings**	1	**0.234** <0.001 **	0.069 0.160	0.057 0.252	0.033 0.505	**0.181** <0.001 **	**0.217** <0.001 **	0.042 0.393	−0.081 0.100
**AT Index**	0.234 <0.001	1	**0.143** 0.004 **	**0.175** <0.001 **	**0.106** 0.032 *	**0.767** <0.001 **	**0.282** <0.001 **	0.023 0.642	**−0.433** <0.001 **
**TG/HDL**	0.069 0.160	0.143 0.004	1	**0.525** <0.001 **	**0.898** <0.001 **	**0.250** <0.001 **	**0.328** <0.001 **	−0.070 0.155	**−0.124** 0.012 *
**LDL/HDL**	0.057 0.252	0.175 <0.001	0.525 <0.001	1	**0.383** <0.001 **	**0.275** <0.001 **	**0.136** 0.006 **	−0.085 0.084	**−0.195** <0.001 **
**TyG Index**	0.033 0.505	0.106 0.032	0.898 <0.001	0.383 <0.001	1	**0.223** <0.001 **	**0.333** <0.001 **	−0.040 0.424	−0.075 0.128
**TyG/ALT Index**	0.181 <0.001	0.767 <0.001	0.250 <0.001	0.275 <0.001	0.223 <0.001	1	**0.134** 0.006 **	**−0.138** 0.005 **	**−0.679** <0.001 **
**HOMA-IR Index**	0.217 <0.001	0.282 <0.001	0.328 <0.001	0.136 0.006	0.333 <0.001	0.134 0.006	1	**0.214** <0.001 **	**0.101** 0.041 *
**Prolactin**	0.042 0.393	0.023 0.642	−0.070 0.155	−0.085 0.084	−0.040 0.424	−0.138 0.005	0.214 <0.001	**1**	**0.779** <0.001 **
**Prolactin/ALT Index**	−0.081 0.100	−0.433 <0.001	−0.124 0.012	−0.195 <0.001	−0.075 0.128	−0.679 <0.001	0.101 0.041	0.779 <0.001	1

ALT: Alanine aminotransferase, AST: Aspartate aminotransferase, AT index (ALT/AST): Alanine aminotransferase/aspartate aminotransferase ratio, Glucose: Fasting plasma glucose, HDL: High-density lipoprotein, HOMA-IR: Homeostasis model assessment of insulin resistance, Insulin: Fasting insulin level, LDL: Low-density lipoprotein, LDL/HDL: Low-density lipoprotein/high-density lipoprotein ratio, MASLD: Metabolic dysfunction-associated steatotic liver disease, TyG index: Triglyceride-glucose index. * *p* < 0.05, ** *p* < 0.01. Spearman’s rho (ρ).

**Table 3:** ROC Analysis Results of Biomarkers for Discriminating MASLD Status.

Index	AUC	*p*	95% CI (Lower)	95% CI (Upper)	Cut-Off Value	Positive LR	Negative LR	Specificity	Sensitivity	Youden Index
**AT Index**	0.621	**<0.001**	0.572	0.668	>0.88	1.43	0.58	0.5069	0.7047	0.2116
**TG/HDL**	0.528	0.330	0.478	0.577	>2.79	1.32	0.87	0.7051	0.3886	0.0936
**LDL/HDL**	0.524	0.397	0.475	0.574	>2.25	1.20	0.86	0.5899	0.4922	0.0820
**TyG**	0.515	0.610	0.465	0.564	>4.41	1.08	0.85	0.3456	0.7047	0.0502
**TyG/ALT**	0.592	**0.001**	0.542	0.640	>78.22	1.36	0.68	0.5300	0.6373	0.1673
**HOMA-IR**	0.610	**<0.001**	0.561	0.657	>4.37	1.58	0.69	0.6498	0.5544	0.1042
**Prolactin**	0.518	0.530	0.468	0.567	>5.75	1.07	0.60	0.1475	0.9119	0.0593
**Prolactin/ALT**	0.541	0.149	0.492	0.590	≤0.72	1.13	0.80	0.3963	0.6839	0.0802

ALT: Alanine aminotransferase, AST: Aspartate aminotransferase, AT index (ALT/AST): Alanine aminotransferase/aspartate aminotransferase ratio, Glucose: Fasting plasma glucose, HDL: High-density lipoprotein, HOMA-IR: Homeostasis model assessment of insulin resistance, Insulin: Fasting insulin level, LDL: Low-density lipoprotein, LDL/HDL: Low-density lipoprotein/high-density lipoprotein ratio, MASLD: Metabolic dysfunction-associated steatotic liver disease, TyG index: Triglyceride-glucose index.

**Table 4:** Multivariate Regression Analysis Results of Indices with Predictive Power in the Diagnosis of MASLD.

Variable	β (B)	SE	Wald	*p*	OR (Exp(B))	95% CI
AT index	1.224	0.349	12.320	**<0.001**	**3.401**	1.717–6.735
BMI SDS	0.557	0.150	13.818	**<0.001**	**1.745**	1.301–2.340
HOMA-IR	0.032	0.031	1.051	0.305	1.033	0.971–1.099
Prolactin/ALT	0.245	0.142	2.974	0.085	1.278	0.967–1.689
Constant	−3.258	0.591	30.370	<0.001	0.038	—

CI: Confidence Interval; OR: Odds Ratio; SE: Standard Error, ALT: Alanine aminotransferase, AST: Aspartate aminotransferase, AT index (ALT/AST): Alanine aminotransferase/aspartate aminotransferase ratio, HOMA-IR: Homeostasis model assessment of insulin resistance, BMI SDS: Body mass index standard deviation score. Omnibus test: χ^2^ = 35.503, *p* < 0.001; Hosmer–Lemeshow test: χ^2^ = 8.319, df = 8, *p* = 0.403; Nagelkerke R^2^ = 0.211; correct classification rate: 62.2%.

## Data Availability

The data presented in this study are available from the corresponding author upon reasonable request. The data are not publicly available due to privacy and ethical considerations.

## Abbreviations

ALT	Alanine aminotransferase
AST	Aspartate aminotransferase
AT index	Alanine aminotransferase/aspartate aminotransferase ratio
AUC	Area under the curve
BMI SDS	Body mass index standard deviation score
CI	Confidence interval
fT4	Free thyroxine
HbA1c	Hemoglobin A1c
HDL	High-density lipoprotein
HOMA-IR	Homeostasis model assessment of insulin resistance
LDL	Low-density lipoprotein
LR	Likelihood ratio
MASLD	Metabolic dysfunction-associated steatotic liver disease
OR	Odds ratio
ROC	Receiver operating characteristic
SD	Standard deviation
TG	Triglyceride
TG/HDL	Triglyceride/high-density lipoprotein ratio
TSH	Thyroid-stimulating hormone
TyG	Triglyceride-glucose
USG	UltrasonographyReferences
